# Visits to Private Asylums

**Published:** 1902-12-27

**Authors:** 


### VISITS TO PRIVATE ASYLUMS.

By Our Rambling Reporter.

### THE WITHAM PRIVATE ASYLUM.

Witham is a pleasant little town in Essex of about 4,000 inhabitants. It is situated on the main London road, and even yet shows some signs of the ancient glory of those times when the arrival and departure of the four-horse coach were the events of the day. In the main street is a fine Georgian residence occupied by Dr. Payne, the present proprietor of the asylum; and lying around the house are 13 acres of land, at one side of which the asylum proper is placed. Some of this land is under grass, and Dr. Payne is thus able to produce all the dairy produce required in the asylum. The rest of the land is laid out in lawns, shrubberies and kitchen gardens. Although literally in the town of Witham the asylum is as much secluded as if it were miles in the country; and it has the great advantage of being within a few minutes' walk of a main-line station on the Great Eastern Railway, about seventy minutes' run from London.

The building is licensed for 26 patients. Many of those in residence at present are old cases who have been in the asylum for a long time; one over 40 years, and two other cases are upwards of 80 years of age. Throughout the whole house there was an air of contentment among the patients, and evident signs of kind and careful attention on the part of the staff.

The asylum is emphatically of the private dwelling-house type, and has very little about it to remind us of the institution. As a quiet home for the insane it is extremely suitable. The bedrooms are nearly all adapted for one patient only, but some of the rooms have two beds, so that when necessary the second one can be occupied by an attendant or nurse, ensuring constant supervision. Private sitting-rooms can be had.

The scale of payments ranges from £60 to £160 a year, and for these sums the accommodation provided is wonderfully good. Of course, patients are admitted at higher payments in cases where special accommodation and extra attendance are wished for.

